# Bioremoval of Different Heavy Metals by the Resistant Fungal Strain *Aspergillus niger*

**DOI:** 10.1155/2018/3457196

**Published:** 2018-11-01

**Authors:** Ismael Acosta-Rodríguez, Juan F. Cárdenas-González, Adriana S. Rodríguez Pérez, Juana Tovar Oviedo, Víctor M. Martínez-Juárez

**Affiliations:** ^1^Universidad Autónoma de San Luis Potosí, Facultad de Ciencias Químicas, Centro de Investigación y de Estudios de Posgrado, Laboratorio de Micología Experimental, Av. Dr. Manuel Nava No. 6, Zona Universitaria, C.P. 78320, San Luis Potosí, Mexico; ^2^Unidad Académica Multidisciplinaria Zona Media, Universidad Autónoma de San Luis Potosí, Carretera San Ciro de Acosta Km. 4, Ejido Puente del Carmen. C.P. 79617, Río Verde, San Luis Potosí, Mexico; ^3^Área Académica de Medicina Veterinaria y Zootecnia, Instituto de Ciencias Agropecuarias, Universidad Autónoma Del Estado de Hidalgo, Zona Universitaria, Rancho Universitario Km 1. C.P. 43600, Tulancingo de Bravo Hidalgo, Mexico

## Abstract

The objective of this work was to study the resistance and removal capacity of heavy metals by the fungus *Aspergillus niger*. We analyzed the resistance to some heavy metals by dry weight and plate: the fungus grew in 2000 ppm of zinc, lead, and mercury, 1200 and 1000 ppm of arsenic (III) and (VI), 800 ppm of fluor and cobalt, and least in cadmium (400 ppm). With respect to their potential of removal of heavy metals, this removal was achieved for zinc (100%), mercury (83.2%), fluor (83%), cobalt (71.4%), fairly silver (48%), and copper (37%). The ideal conditions for the removal of 100 mg/L of the heavy metals were 28°C, pH between 4.0 and 5.5, 100 ppm of heavy metal, and 1 g of fungal biomass.

## 1. Introduction

Heavy metals are ubiquitous contaminants that have accompanied the man from the earliest ancient times, and unlike other environmental pollutants, heavy metals are chemical elements that man does not create or destroy. The role that man plays in the environmental presence of metals is to introduce into the environment these elements as a result of different human activities, and on the other hand, to alter the chemical or biochemical form in which they are. Metals are naturally subjected to biogeochemical cycles that determine their presence and concentration in different natural environments such as soil, groundwater and surface, air, and living beings. Human intervention can greatly modify the concentration of metals in these environments and facilitate their distribution from the mineral reserves in which metals are naturally confined [1]. From the toxicological point of view, metals often present a marked multiplicity of toxic effects. The specific chemical species of the metal strongly influences these effects, as well as the toxicokinetic variables of absorption, distribution, and excretion. The toxicological significance of heavy metals, taking into account their ubiquity, the extent of their industrial and domestic uses, as well as their environmental persistence, which has to be evaluated based on the characteristics of the chemical compound of which the metal is part, and which determine its mobility environmental and its bioavailability [2].

In Mexico, there are reports of the presence of heavy metals in rivers, lakes, crops, soils, and air urban areas, as well as in coastal environments and marine ecosystems, where the accumulation of toxic metals in fish and shellfish tissues of human consumption is seen [3, 4], and mining is one of the main causes of environmental pollution by heavy metals, mainly due to the inadequate management of their so-called mine tailings. There are reports of widespread contamination in states of the Mexican Republic such as Zacatecas, San Luis Potosí, Guerrero, and Sonora. [4–6]. Reports indicate that in Mexico there could be million tons of pull at the national level, of which they are still unknown the conditions and their potential to affect the environment [1, 7]. The most common example is the soil contamination, which occurs during the extraction of gold and silver, commonly made by amalgamation with mercury and cyanidation. In neither case, there is a total recovery of the compounds or added elements, so it is common to find them in the process residues (mining sludges) in the soluble form [1, 4, 7], so that, the “tailings” contain a great quantity of residual metals that derive from a process of extraction that is not 100% efficient, so they exceed the maximum permissible limits of these metals for soils and waters in Mexico, established in the NOM-147-SEMARNAT/SSA1-2004 [8] and NOM-001-SEMARNAT-1996 [9], respectively. In humans, heavy metals can become very toxic when introduced into the organism. At high concentrations, these can cause skin rashes, stomach upset (ulcers), respiratory problems, weakening of the immune system, damage to the kidneys and liver, hypertension, alteration of genetic material, cancer, neurological disorders, and even death [10]. Despite existing legislation on disposal and waste management, it is clear that the problem persists. The foregoing, together with the waste from mining, aggravates the situation of the contamination by heavy metals in Mexico. Different public institutions have developed investigations to establish the magnitude of this problem and have proposed strategies to contribute to the solution of the same, focusing on the use of biological alternatives that result in a lower alteration of the environment, specifically through the use of microorganisms for the removal of heavy metals or biosorption [11]. There are many reports of the isolation of resistant microorganisms to heavy metals and the use of microbial biomass for the removal of heavy metals, from industrial wastewater and/or contaminated water: the resistance and removal of *Rhizopus stolonifer* to lead, cadmium, copper, and zinc [11], the tolerance and removal mechanisms of heavy metals (lead, cadmium, and chromium), by the fungus *Pleurotus ostreatus* HAAS [12], *Bacillus megaterium* nickel resistance and her capacity of removal [13], heavy metal susceptibility and removal potential (mercury, copper, and lead) of *Rhodotorula mucilaginosa* [14], the resistance of *Alcaligenes* sp. BAPb.1 to lead (II), copper (II), zinc (II), nickel (II), and chromium (VI), and his capacity for removal of lead (II) [15], the isolation and identification of fungi and yeast resistant to lead (II) [16], the resistance and removal of chromium (VI) by *Aspergillus niger* [17], the removal of different heavy metals by *A. niger* [18], the removal of lead, cadmium, copper, and nickel by *A. niger* [19], the removal of aluminum, iron, lead, and zinc by *A. niger* during the bioleaching process [12], and the removal of copper (II), manganese (II), zinc (II), nickel (II), iron (III), lead (II), and cadmium (II) by immobilized cells of *A. niger* [20], with highly satisfactory results. This work reports the removal of different heavy metal in an aqueous solution by a strain of *A. niger* which is highly resistant to some heavy metals.

## 2. Experimental

### 2.1. Microorganisms and Heavy Metals Resistant Tests

The fungal strain of *A. niger* was isolated from the polluted air in a fuel station, near to the Faculty of Chemical Science, belonging to the Autonomous University of San Luis Potosí (San Luis Potosi, Mexico) [17], and this was used for the screening. In addition to the above, this fungus was conditioned for years under conditions of biological stress and was inoculated in culture media containing between 0 and 500 ppm of different heavy metals such as chromium, lead, cadmium, arsenic, etc. For the isolation, growth, and pH calibration, we carried out the methodology of Acosta-Rodríguez et al. [21] as follows: on Petri dishes containing modified Lee's minimal medium (LMM) (with 0.25% KH_2_PO_4_, 0.20% MgSO_4_, 0.50% (NH_4_)_2_SO_4_, 0.50% NaCl, 0.25% glucose, and 2% agar) supplemented with 500 mg/L of K_2_CrO_4_. The pH of the medium was adjusted with a pH meter Corning Pinnacle 540 and maintained at 5.3 with 100 mmol/L of citrate phosphate buffer. The plates were incubated at 28°C for 7 days. Fungal cultures grown in thioglycolate broth were used as primary inoculums. Heavy metals resistant tests of the isolated strain, the fungi *A. niger*, were performed on liquid LMM containing the appropriate nutritional requirements and different concentrations of heavy metals (as salt), and the dry weight was determined.

### 2.2. Heavy Metal Resistance Assay

For the resistance test, we followed the methods of Acosta-Rodríguez et al. [21], where Petri dishes were prepared with Sabouraud Dextrose Agar, added with different heavy metals salts. The prepared plates were inoculated with 1 × 10^6^ spores/mL, uniformly spread throughout the dishes, and incubated at 28°C for 7 days, and the growth of the plates was compared with a control.

### 2.3. Obtaining the Fungal Biomass

For their propagation, 1000 mL Erlenmeyer flasks containing 600 mL of thioglycolate broth (8 g/L) were used. The prepared flasks were inoculated with 1 × 10^6^ spores/mL and were incubated at 28°C, with constant stirring (100 rpm) [21]. After 5 days of incubation, the cells were filtered in Whatman paper No. 1, washed twice with trideionized water, and then dried at 80°C for 12 h in an oven. Finally, the fungal biomass was milled and stored in an amber bottle at room temperature until their use.

### 2.4. Preparation of Iron Oxide-Coated Biomass

80 mL of 2 M Fe(NO_3_)_3_·9H_2_O was prepared and 1.0 mL of 10 M·NaOH was added to this solution and mixed thoroughly. 20 g of the fungal biomass powder was taken in a porcelain pot, and a mixture of iron oxide and NaOH solution was added to the porcelain pot and homogenized, kept in an oven for 3 h at 80°C. Afterwards, the oven temperature was raised to 110°C and continued for 24 h. The coated biomass powder was separated by crushing with mortar and pestle [22].

### 2.5. Biosorption Tests for Heavy Metals by Fungal dry Cells

Solutions of heavy metals for analysis were prepared by diluting 1 g/L of stock metal solution. The concentration range of heavy metals solutions was 1–100 mg/L. The pH of each solution was adjusted to the required value by adding 1 M·H_2_SO_4_ solution before mixing with the fungus with an analyzer Corning Pinnacle 540. The biosorption of the metals by fungal dry cells was determined at different concentrations of 100 mL heavy metal solution, with 1 g of fungal biomass, at 100 rpm, and the sample was filtered, and the supernatant was analyzed for residual heavy metals at different times after a contact period: zinc (II), lead (II), mercury (II), cadmium (II), spectrophotometrically with a Genesys 10S Vis and the dithizone method [23], cobalt (II) by methyl isobutyl ketone [24], fluorine (I) by specific ion, and copper (II), arsenic (III), arsenic (V), and silver (I), by atomic absorption with spectrophotometer Varian SpectrAA 220 [25]. Moreover, biosorption to the contaminated water was examined. To six Erlenmeyer glass flasks add 5 g of fungal biomass and 95 mL of water (263 mg/L of lead (II), 183 mg/L of Mercury (II), and 250 mg/L of cobalt (II)), from the farmland of the “Tanque Tenorio” (which is southeast of the city, in the municipality of Soledad de Graciano Sánchez, S.L.P., Mexico, and is a catchment lagoon of wastewater, of which 60% and 40% are from urban and industrial origin, respectively) (it should be noted that the industrial zone of San Luis Potosí has more than 520 companies, among which are the mining-metallurgists, textiles, and chemicals) [25], and they were incubated during 7 days, stirred at 100 rpm, and filtered in Whatman filter paper No. 1, and the concentration of lead (II), Mercury (II), and zinc (II) of the filtrate was analyzed.

## 3. Results and Discussion

### 3.1. Isolation and Identification of a Fungal Strain Resistant to Heavy Metals

**Figure 1:**
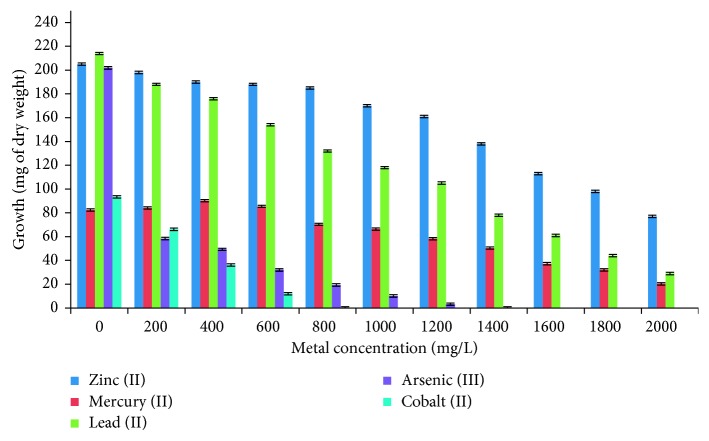
Growth in dry weight of *Aspergillus niger* with different heavy metals concentrations: 1 × 10^6^ spores/mL; 28°C, 7 days of incubation; 100 rpm.

The fungal strain was kept in culture medium like LMM containing different concentrations of heavy metals for many years, which caused mechanisms of adaptation and resistance to these metals, causing the fungus to not die intoxicated and could remove several of them. This indicates that these fungi developed the heavy metal tolerance and/or resistance, and they were identified by their macroscopic and microscopic characteristics [26]. In a previous study, it has been reported that the fungus grew in 2000 mg/L (42 *µ*g of dry weight) of chromium (VI), and it presents very good adsorption capacity of chromium (VI) in different conditions [17]. Also, the strain grew on LMM supplemented with different concentrations of heavy metals, about 37.6%, 24.6%, and 13.5%, of zinc (II), mercury (II), and lead (II), respectively, of growth relative to control without metal, and, therefore, probably is resistant to the metals, although, it grew a 16% with 1.4 g/L of arsenic (III), and it is very sensitive to cobalt (II) (12.8% with 600 mg/L) (Figure 1). On the other hand, in plate-resistant testing, the fungus grows in 2000 mg/L of zinc (II), lead (II), mercury (II), and chromium (VI), 1200 g/L of Arsenic (III), 600 mg/L of cobalt (II), and 400 mg/L of cadmium (II) (Table 1), showing that the fungus has the ability to grow at very high concentrations of these different toxic metals, and it can present different mechanisms of resistance and/or adaptation to toxic metals.

Different microorganisms that are heavy metals resistant have been isolated from different contaminated sites: screening the resistance to lead, cadmium, copper, and zinc of five fungal species isolated from soils: *Emericella quadrilineata*, *A. niger*, *Macrophomina phaseolina*, *R. stolonifer*, and *Aspergillus fumigatus*, and the most resistant fungal species (1 g/L of metals) was *R. stolonifer* followed by *M. phaseolina* which showed resistance with all the metals, while *A. niger, A. fumigatus*, and *E. quadrilineata* were more sensitive to these heavy metals [11], the fungus *P. ostreatus* HAAS grew very well in 500 mg/L of lead, and concentrations of 30 mg/L of cadmium and 200 mg/L of chromium appeared to inhibit the growth of the fungus [12], *B. megaterium* strain MNSH1-9K-1 tolerate up to 200 ppm of each nickel and vanadium [13], *Alcaligenes* sp. BAPb.1, grow in the presence of 1000 mg/L of lead (II), 600 mg/L of copper (II), 600 mg/L of zinc (II), 400 mg/L of nickel (II) and chromium(VI) [15], *Penicillium* sp., *Trichoderma* sp., and *Alternaria* sp., isolated from the farmland of the “Tanque Tenorio”, grow with 500–2000 mg/L of lead (II) [16], *A. niger* has been growing in the presence of different concentrations of metals like nickel, cobalt, iron, magnesium, and manganese [27], and the yeast *Candida tropicalis*, isolated from wastewater from industrial area of Sheikhupura, which is grown in 2.5 g/L of cadmium (II), zinc (II) (1.4 g/L), nickel (II) (1 g/L), Mercury (II) (1.4 g/L), copper (II) (1 g/L), chromium (VI) (1.2 g/L), and lead (II) (1 g/L) [28].

### 3.2. Removal of Different Heavy Metals by Fungal Biomass of *A. niger*

On the other hand, we analyzed the capacity of heavy metals removal by dry cell of the fungus. The results are shown in Table 2. The fungus removed efficiently most of the heavy metals analyzed: zinc (II) (100%), mercury (II) (83.2%), fluor (I) (83%), and cobalt (II) (71.4%), and less efficiently: silver (I) (48%) and copper (37%). Dead fungal cells can be effective metal accumulators, and there is evidence that some biomass-based cleanup processes are economically viable [2, 4]. The tolerance of some fungal species to heavy metals, as well as the physiological response to them, has been also determined [2, 11–13]. The removal of heavy metal ions, using fungus as biosorbents, was previously investigated [2, 4, 12, 17–20]. Our results confirm the capacity of the microorganisms biomass for the removal of heavy metals with different effectivity, like bacteria, fungus, yeast, and algae-based microbiological decontamination of heavy metals contaminated soils of different places [2–5]: *M. phaseolina* and *R. stolonifer* for the removal of lead, cadmium, copper, and zinc, from soil [11], the removal of lead, cadmium, and chromium, in liquid culture with *P. ostreatus* HAAS [12], the removal of lead, cadmium, copper, and nickel, with *A. niger* [19], the elimination of copper, cadmium, lead, and zinc in dried soil residues with *A. niger* during the bioleaching process [12], the removal of copper (II) and cadmium (II) in batch systems by immobilized cells of *A. niger* [20], the removal of 90% of chromium (VI) by NaOH-pretreated *A. niger* biomass, and that heavy metal uptake by live *A. niger* biomass for cadmium (II) and for zinc (II) [29], yeasts isolated from water, soil, and plant environments [30], and other studies with other species of fungi [4, 18, 19, 31–34]. According to our results, we can assume the surface of the biomass coated with iron oxide is partially ionized, causing the pH to approach neutrality; apparently, the OH^−^ groups present compete for the binding sites with the heavy metals and the biomass, decreasing the removal of metals, while at acid pH, there is a better removal of the heavy metals [35].

### 3.3. Removal of Heavy Metals in Industrial Wastes with Fungal Biomass

**Figure 2:**
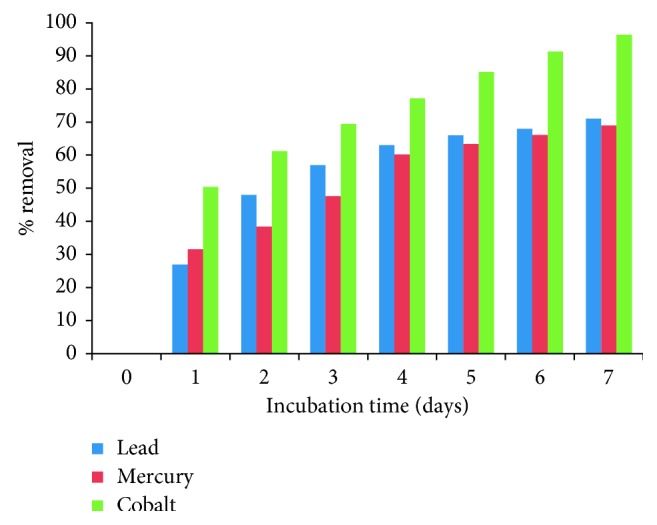
Removal of heavy metals from industrial wastes incubated with 5 g of fungal biomass and 95 mL of water contaminated with 263 mg/L of lead (II), 183 mg/L of mercury (II), and 250 mg/L of cobalt (II), at pH 5.0 (adjusted), 28°C, and 100 rpm.

For analyzing the possible use and the ability of *A. niger* biomass to removal of lead (II), cobalt (II), and mercury (II), from wastewater, a removal assay was mounted in an aqueous solution in the presence of 5 g biomass, with 95 mL of nonsterile water contaminated (from “Tanque Tenorio”) with 263 mg/L of lead (II), 183 mg/L of mercury (II), and 250 mg/L of cobalt (II), at pH 5.0 (adjusted), 28°C and stirring at 100 rpm. It was observed that at 7 days of incubation, 71%, 69%, and 96.4%, of the heavy metals present in the water contaminated were removal, respectively (Figure 2). The metal removal capability by the biomass of *A. niger* is equal to or greater than the other biomasses that have been studied, like the removal of mercury, cadmium, an copper (4.79%, 10.25%, and 5.49%, respectively), using *R. mucilaginosa* planktonic cells during 48 hours [17], the metal removals during two-step process using *A. niger* reached 84.3%, 84.4%, 25%, and 14.4% for copper, cadmium, lead, and zinc, respectively [12], the removal of cadmium (II) (95%), lead (II) (88%), iron (III) (70%), copper (II) (60%), nickel (II) (48.9%), manganese (II) (37.7%), and zinc (II) (15.4%), from industrial wastewater in batch systems by immobilized cells of *A. niger* [20], the use of the extracellular media of *Alternaria alternata-*containing organic acids and siderophores for the metal leaching (vanadium, aluminum, molybdenum, magnesium, iron, nickel, arsenic, and chromium) [32], the removal of 67% of arsenic (III) from samples of groundwater contaminated with 1 mg/L from the metalloid, coming from Zimapan, Hidalgo's state, Mexico [36], the 99.35% removal of copper with pure and modified chitosan hydrogels from shrimp shell, from copper leachate [37], *Saccharomyces cerevisiae* and *Torulaspora delbrueckii* decrease in 98.1%, 83.0%, 60.7%, 60.5%, and 54.2% for turbidity, sulphates, BOD, phosphates, and COD, respectively, of the tannery effluent [38], *C. tropicalis* removed 40% of cadmium (II) from the wastewater after 6 days and was also able to remove 78% from the wastewater after 12 days [28], and *S. cerevisiae* “wild-type” (WT) parental strain BY4741, very efficient in removing manganese (II), copper (II), and cobalt (II) from synthetic effluents containing 1-2 mM cations [39]. Industrial effluents often contain more than one type of metal ion; these may interfere in the removal/recovery of the metal ion of interest. Limited information about the effect of cocations is available in the literature. The presence of other cations (cocations) can affect the sorption of metal ions (primary cation) to the biomass, and in some cases, it may affect the removal efficiency [40].

## 4. Conclusion

We isolated a fungus, which was identified such as *A. niger*, which grow with different heavy metals in LMM, and probably is resistant to the metals. The dead fungal biomass removed efficiently different heavy metals (chromium (VI) and zinc (II) (100%), mercury (II) (83.2%), and fluorine (I) (83%)) at different pH conditions (4.0 for lead (II), 5.0 for zinc (II), and cobalt (II), and 5.5 for mercury (II)), 28°C, and 1 g of fungal biomass. Finally, these results suggest the potential applicability of this fungus for the remediation of heavy metals from polluted soils and waters.

## Figures and Tables

**Table 1:** Growth in LMM in plate of *Aspergillus niger* with different heavy metals: 1 × 10^6^ yeast/mL, 28°C, and 7 days of incubation.

Heavy metal	Growth Heavy metal concentration (mg/L)
Zinc (II)	2000
Lead (II)	2000
Mercury (II)	2000
Chromium (VI)	2000
Arsenic (III)	1200
Arsenic (V)	1000
Copper	1000
Silver	1000
Fluor	800
Cobalt	600
Cadmium	400

**Table 2:** Removal of different heavy metals by fungal biomass of *A. niger*: 28°C, 1 g of fungal biomass, 100 rpm, 24 h.

Heavy metals	pH	Initial concentration (mg/L)	% removal
Chromium (VI)	1.0	50	100^*∗*^
Zinc (II)	5.0	100	100^*∗∗*^
Mercury (II)	5.5	100	83.2
Fluor (I)	6.0	10	83.0
Cobalt (II)	5.0	100	71.4
Arsenic (V)	6.0	1.0	69^*∗∗∗*^
Arsenic (III)	6.0	1.0	66^*∗∗∗*^
Lead (II)	4.0	100	59.0
Cadmium (II)	6.0	5.0	57.0
Silver (I)	6.0	100	48.0
Copper (I)	5.0	100	37.0

^*∗*^30 minutes [17]. ^*∗∗*^165 minutes. ^*∗∗∗*^Fungal biomass modified with Fe(NO_3_)_3_·9H_2_O.

## Data Availability

The figures (graphics) and tables or any information data used to support the findings of this study are included within the article, and all information used to support the findings of this study are available from the corresponding author upon request.
